# Degradation of Methyl Blue Using Fe-Tourmaline as a Novel Photocatalyst

**DOI:** 10.3390/molecules18021457

**Published:** 2013-01-24

**Authors:** Xuesen Bian, Jianqiu Chen, Rong Ji

**Affiliations:** 1 State Key Laboratory of Pollution Control and Resource Reuse, School of the Environment, Nanjing University, Nanjing 210023, China; 2 Nanjing Institute of Environmental Sciences of the Ministry of Environmental Protection of China, Nanjing 210042, China; 3 Department of Environmental Science & Department of Analytical Chemistry, China Pharmaceutical University, Nanjing 210009, China

**Keywords:** tourmaline, methyl blue, photocatalysis, affecting factors

## Abstract

This study investigated the photocatalytic activity of tourmaline by itself. Under irradiation of a 13 W, 254 nm UV lamp, 50% of methyl blue disappeared in the presence of 130 mg·L^−1^ tourmaline. The reaction was inhibited by the addition of ethanol, Cl^−^, SO_4_^2−^ and Cu^2+^, and promoted a little by addition of 50 mg/L Mg^2+^, which supports the inference of involvement of ^•^OH radicals. This is the first proposal of tourmaline as a single photocatalyst without the need to add any artificial chemical products. Results from this study might contribute to the broadened usage of this mineral to approach the goals of saving energy and eliminate direct or indirect environmental pollution.

## 1. Introduction

Wastewater from the dye industry is characterized by complex composition [1], high visibility [2] and obvious toxicity [3], thus representing a pollution source deserving special attention [4]. It’s estimated that some 600–700 million tons of dye wastewater are released annually into the environment, which represents 10 percent or so, of the total effluents in China [5]. In recent years, lots of efforts have been devoted to the treatment of dye wastewaters, which are trying to remove such pollutants by adsorption, active sludge oxidization, ion exchange or ozone treatment. However, such methods often cannot meet all requirements generally considered necessary in wastewater processing, *i.e.*, complete decomposition and detoxification of target contaminants, effective removal of COD as well as satisfactory processability of highly polluted wastewaters. Photocatalysis is a novel method which has attracted a lot of interest in the last several decades [6], due to its innocuity and low cost, as well as its wide applicability for a large variety of pollutants.

Tourmaline is a natural mineral widely distributed in China. The special crystal structure of its trigonal system makes it able to generate a spontaneous electric field within, in which electrons can be driven to flow continually from the negative to the positive pole. The spontaneous field of this mineral makes it useful in various areas of environmental protection: the electric field continually generates anions in the surrounding air, which helps reduce various pollutants like nitrogen oxides, oxygen radicals, *etc.* The interfacial activity makes it able to act as a cleaning agent instead of detergents. When it is used in water, the facial hydroxyls around it can adsorb contaminant metallic ions [7]. Water pH can be adjusted by tourmaline through its electric pole and ion exchange effects [8]. In the field of photochemical research, it was found that this mineral can act as the iron source for Fenton reagent to enhance the promotion of photodegradation of Argazol blue [9] and Orange II [10] by H_2_O_2_. Usage of H_2_O_2_ can be regarded as a method of introduction of little additional pollution and thus less hazardous to the environment in some ways, but the production of this chemical itself usually generates various pollutants, which require further treatment. This study is designed to initially investigate the photocatalytic activity of tourmaline mineral by itself without any addition of artificial chemical products, using simulated methyl blue (MB) wastewater as the treatment target.

## 2. Results and Discussion

### 2.1. Photocatalytic Degradation of MB by Fe-Tourmaline Powder

**Figure 1:**
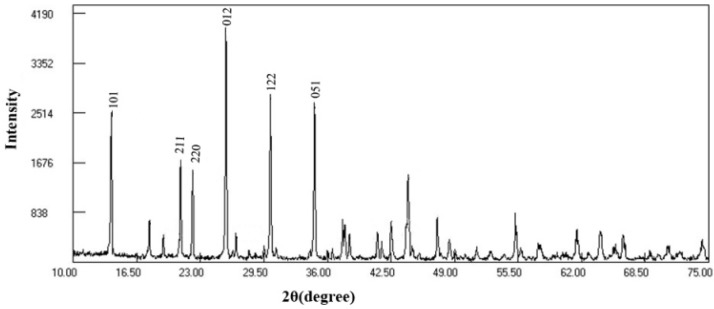
XRD spectrum of tourmaline used in this study.

The XRD result of Fe-tourmaline used in this study is shown in Figure 1. The 2θ values of 13.88°, 20.96°, 22.20°, 30.14° and 34.66° are in accord with those reported by Meng *et al*. [11], demonstrating the characteristic peaks of Fe-tourmaline. The XRF analysis further showed that the tourmaline powder contains in weight, 48.91% of SiO_2_, 39.52% of Al_2_O_3_, 5.41% of Fe_2_O_3_, 2.36% of CaO, 1.38% of K_2_O, 1.13% of Na_2_O, 1.29% of MgO.

**Figure 2:**
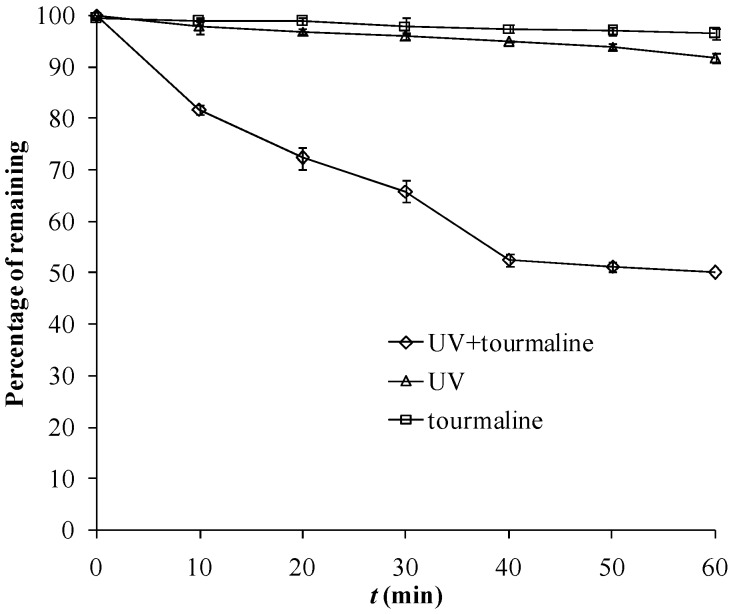
Photocatalytic degradation of 11 mg·L^−1^ MB in ultrapure water under UV 254 nm (13 W) as air was pumped in at 1 mL·s^−1^, and control experiments without UV or tourmaline.

As shown in Figure 2, MB disappeared by 8% with only UV irradiation. As a common biological stain, it should be stable enough to light. With the addition of tourmaline, the degradation of MB was obviously accelerated. Addition of 3000-mesh tourmaline in the UV photodegradation system resulted in an increase of the MB disappearance rate by 50%. Additionally, no adsorption occurred during this process (Figure 2). The disappearance should only be ascribed to photocatalytic degradation by the tourmaline mineral. To our knowledge, this is the first discovery and proposal of the photocatalytic effects of tourmaline alone.

### 2.2. Effect of Ethanol

**Figure 3:**
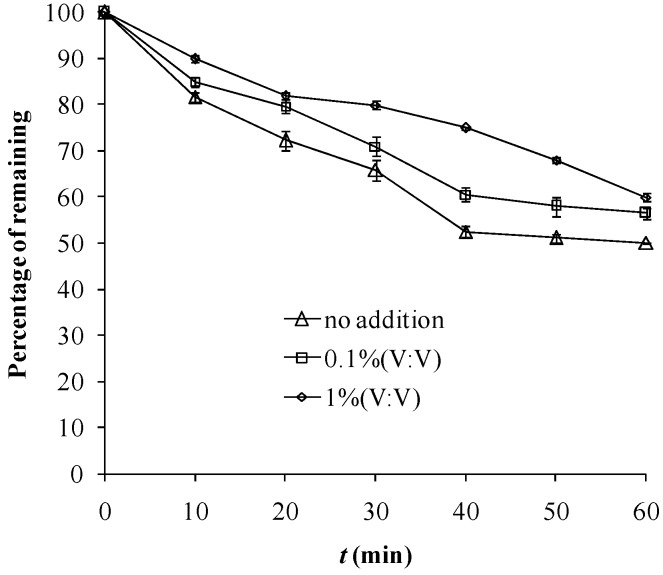
Photocatalytic degradation of 11 mg·L^−1^ MB in ultrapure water under UV 254 nm (13 W) as air was pumped in at 1 mL·s^−1^, with or without ethanol addition.

As shown in the XRF results, elemental iron exists in the Fe tourmaline used in this study, mainly in the form of Fe_2_O_3_. Photocatalytic degradation catalyzed by Fe mineral was ascribed to three different mechanisms [12], *i.e.*, that of photoinduced ligand-to-metal charge transfer (LMCT), of semiconductor photocatalysis and of photo-Fenton reactions through the generation and participation of H_2_O_2_. Organic alcohols like methanol, ethanol, *t*-butanol, *etc.*, are typically scavengers of hydroxyl radicals [13]. Ethanol addition exhibited depressing effects for this reaction, as shown in Figure 3, which might support the involvement of ^•^OH radicals. Photocatalytic reactions through a ^•^OH-based mechanism were also reported for some other Fe-containing minerals [14,15]. In a photocatalytic system, Cl^−^ may act as a competitor of catalyst adsorption sites or scavenger of ^•^OH [16]:

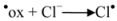



### 2.3. Effect of Cl^−^ and SO_4_^2−^

**Figure 4:**
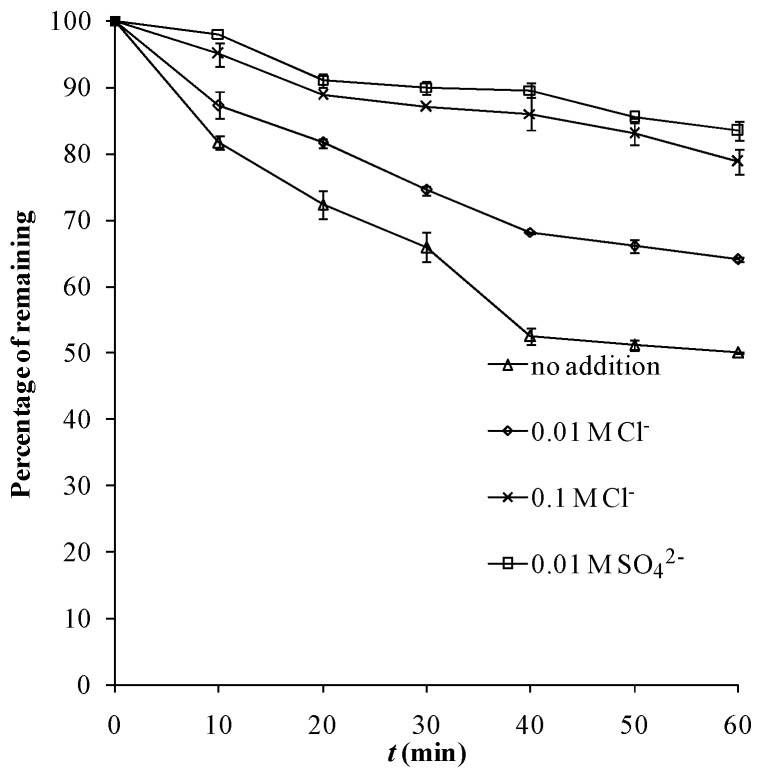
Photocatalytic degradation of 11 mg·L^−1^ MB in ultrapure water under UV 254 nm (13 W) as air pumped in at 1 mL·s^−1^, with or without the addition of Cl^−^ or SO_4_^2−^.

In this study, almost no adsorption seemed to occur between MB and tourmaline powder (Figure 2), thus the adsorption competition can be excluded. In Figure 4, presence of both levels of Cl^−^ depressed the photocatalytic reaction, with the stronger inhibition corresponding to the higher level. This phenomenon might be ascribed to the ^•^OH scavenging effect of Cl^−^ in solution.

The addition of SO_4_^2−^ inhibited the reaction, too. SO_4_^2−^ in a photocatalytic system can also act as the scavenger of ^•^OH radicals [17]:
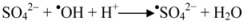


At the same level, the depressing extent of SO_4_^2−^ was obviously higher than that of Cl^−^ (Figure 4), which is opposite to the results reported by reference [17]. The product of ^•^SO_4_^2−^ can also react with the substrate, which helps reduce the inhibition effect. The stronger extent of inhibition by SO_4_^2−^ in this study might be ascribed to the different reaction rates of ^•^SO_4_^2−^ with various substrates.

### 2.4. Effect of Cu^2+^ and Mg^2+^

**Figure 5:**
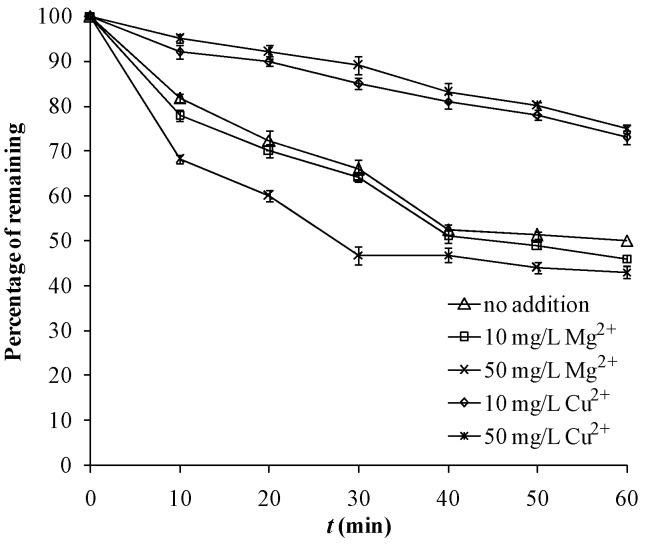
Photocatalytic degradation of 11 mg·L^−1^ MB in ultrapure water under UV 254 nm (13 W) as air is pumped in at 1 mL·s^−1^, with or without the addition of Mg^2+^ or Cu^2+^.

The effects of Cu^2+^ on a ^•^OH-mediated heterogeneous photocatalytic system might not be universal. Some researchers have reported an accelerating effect [18,19], due to a mechanism whereby photogenerated electrons are absorbed by the d orbit holes of copper, and the inhibition of electron-hole recombination will result in more holes reacting with the substrates, but some authors found that Cu^2+^ slowed down the reaction rate when it reached a certain level [19]. The mechanism might be described as one where Cu^2+^ can scavenge the photogenerated holes at the surface leading to the suppression of oxidative reaction; Cu^2+^ might be combined with the organic substrate to form a complex and inhibit the reaction; the conjugation of Cu^2+^ with photoelectrons might reduce the ^•^OH radicals formed through the reaction of oxygen with electrons. In this study, the presence of 10 mg·L^−1^ and 50 mg·L^−1^ Cu^2+^ obviously inhibited this tourmaline-photocatalyzed reaction (Figure 5). Ten mg·L^−1^ Mg^2+^ did not significantly affect the reaction (Figure 5), but when the level of Mg^2+^ reached 50 mg·L^−^^1^, the removal rate was enhanced a bit. This might be due to its absorption of photoelectrons to result in the acceleration of the photoreaction.

## 3. Experimental

### 3.1. General

Methyl blue (MB) was bought from Sigma-Aldrich (St. Louis, MO, USA). The HPLC mobile phase was of chromatographic grade, while other chemicals were analytical grade, and used without further purification. Fe-tourmaline was purchased from Chuanshi Mineral Processing Factory (Hebei, China). The photoreactor was designed according to Jiang *et al*. [20]. The 13 W high-pressure mercury lamp emitting monochromatic light at 254 nm used in our experiments was purchased from the Xujiang Electromechanical Plant (Nanjing, China). The HPLC system used in this study was an Agilent 1200 series HPLC system, consisting of a G1322 A degasser, a G1311 A Quat Pump, a G1329 A thermostatted autosampler and a G1316A column oven. The column for separation was an Agilent Eclipse XDB-C18 (5 μm, 4.6 × 250 mm). The X-ray diffractometer was from X’TRA, with CuKα irradiation. The X-ray fluorescence spectrometer was an XRF-1800 (Shimadzu Co., Kyoto, Japan).

### 3.2. Photocatalytic Degradation of MB by Crude Fe-Tourmaline

About 11 mg·L^−1^ MB solution was prepared by dissolving 5.5 mg MB into 500 mL Milli-Q ultrapure water, which was then transferred into the quartz reactor. After that, chemicals for investigation of effects of affecting factors were introduced in (for the effects of ions, sodium salts were used for anions and chloride salts for cations), and 130 mg·L^−1^ Fe-tourmaline powder was added. The first sample was taken after 1 h of magnetic stirring of the suspension. The high-pressure mercury lamp was inserted in the hollow of the reactor, with a distance between the lamp and the photoreactor surface of 0.015 m. After equilibrium of the suspension, the lamp was turned on, and photocatalytic degradation was started. All experimental processes were accompanied by vigorous stirring and air-pumping into the system at 1 mL·s^−1^. Samples were extracted by a 10-mL injector per internal of 10 min. The sample was then centrifuged (16,000 *g*, 10 min) and the supernatants separated to be stored under −20 °C until HPLC analysis. All experiments were performed in duplicate and at room temperature. The control experiments, without UV irradiation or tourmaline powder, were carried out according to the same procedures.

### 3.3. HPLC Analysis and Tourmaline Characterization

The mobile phase used for the analysis of MB was acetonitrile-water = 40:60 (v:v) isocratically running at a rate of 1 mL·min^−1^. The characterization of tourmaline was under the following conditions: the tube voltage and current were 40 kV and 40 mA, respectively. The 2-θ was measured from 10 to 70 degrees with a step size of 0.02 degree and scan rate of 10 s·step^−1^.

## 4. Conclusions

Tourmaline can act as a catalyst for the photocatalytic UV degradation of methyl blue. The primary results showed that the reaction might be mainly through a ^•^OH-mediated system. This study is the first to use tourmaline mineral alone as the photocatalyst, the catalytic effect of which needs no pretreatment or addition of other artificially synthesized chemicals. This technology promises a broad utilization in the future.
